# CATCHprofiles: Clustering and Alignment Tool for ChIP Profiles

**DOI:** 10.1371/journal.pone.0028272

**Published:** 2012-01-04

**Authors:** Fiona G. G. Nielsen, Kasper Galschiøt Markus, Rune Møllegaard Friborg, Lene Monrad Favrholdt, Hendrik G. Stunnenberg, Martijn Huynen

**Affiliations:** 1 Centre for Molecular and Biomolecular Informatics, Radboud University Nijmegen Medical Centre, Nijmegen, The Netherlands; 2 Department of Mathematics and Computer Science, University of Southern Denmark, Odense, Denmark; 3 Molecular Biology, Faculty of Science, Nijmegen Centre for Molecular Life Sciences, Nijmegen, The Netherlands; 4 eScience Centre, University of Copenhagen, Copenhagen, Denmark; Massachusetts General Hospital, United States of America

## Abstract

Chromatin Immuno Precipitation (ChIP) profiling detects *in vivo* protein-DNA binding, and has revealed a large combinatorial complexity in the binding of chromatin associated proteins and their post-translational modifications. To fully explore the spatial and combinatorial patterns in ChIP-profiling data and detect potentially meaningful patterns, the areas of enrichment must be aligned and clustered, which is an algorithmically and computationally challenging task. We have developed CATCHprofiles, a novel tool for exhaustive pattern detection in ChIP profiling data. CATCHprofiles is built upon a computationally efficient implementation for the exhaustive alignment and hierarchical clustering of ChIP profiling data. The tool features a graphical interface for examination and browsing of the clustering results. CATCHprofiles requires no prior knowledge about functional sites, detects known binding patterns “ab initio”, and enables the detection of new patterns from ChIP data at a high resolution, exemplified by the detection of asymmetric histone and histone modification patterns around H2A.Z-enriched sites. CATCHprofiles' capability for exhaustive analysis combined with its ease-of-use makes it an invaluable tool for explorative research based on ChIP profiling data.

CATCHprofiles and the CATCH algorithm run on all platforms and is available for free through the CATCH website: http://catch.cmbi.ru.nl/.

User support is available by subscribing to the mailing list catch-users@bioinformatics.org.

## Introduction

Chromatin Immuno Precipitation (ChIP) profiling techniques detect *in vivo* protein-DNA binding. The DNA bound by the protein of interest is co-immunoprecipitated using protein-specific antibodies (ChIP), and mapped to the genome either using a DNA microarray chip (ChIP-on-chip) or by sequencing (ChIP-seq), for a review see Collas *et al*.

ChIP profiling has been used not only to detect *in vivo* transcription factor binding sites [1]–[5] but also to map the epigenetic profile of the chromatin, e.g. histone occupancy and histone modifications [6]–[9]. ChIP profiling has revealed a high complexity of binding patterns, both for transcription factor binding sites and for epigenetic markers. The DNA-binding proteins show temporal variation in binding [9], [7], [10], as well as a combinatorial variation over different binding sites in the genome [11]. The various combinations of histone modifications are thought to instruct the cellular machinery [12] while the combinatorial presence of transcription factors could provide a mechanism to exert complex gene regulation [13].

The initial analysis of ChIP-profiling data is primarily concerned with detecting the binding sites in the genome and correlating regions that have specific combinations of chromatin modifications with other observables like gene expression. Such an exploration of the biological relevance of the spatial and temporal combinations of DNA-binding proteins and their modifications requires the clustering of similar ChIP profile regions. One approach is to discretize the data to a simple presence/absence call of each ChIP signal per region, and then classify regions by their binary presence/absence combinations [14], [15]. However, this approach does not exploit the rich information of the individual peak height, width, nor of the variation in signal shapes and relative positions within the regions. Another approach is to compile sets of genomic regions with similar annotated functions and determine their average ChIP profile signal pattern. This approach is easy to apply but does not allow the exploration of new patterns in unannotated regions. In general, a major challenge in the clustering of ChIP profiling patterns is to compare and cluster binding profiles to enable further analysis of the identified clusters without *a priori* binning genomic locations of known functions such as transcription start sites, or reducing the complexity of the data by not including the relative positions and shapes of the ChIP profiling signals. Not only does this call for an unsupervised clustering method that can manage high-resolution ChIP profiling data, it also requires the method to account for the unknown relative positioning of novel patterns, necessitating the alignment of the ChIP profile regions. Furthermore, it requires a flexible organization and graphical presentation of the results to allow browsing and selecting the results for further analysis.

To meet this challenge we have developed the CATCH (Clustering and AlignmenT of ChIp profiles) algorithm and implemented it in the tool CATCHprofiles. The CATCH algorithm is designed to handle ChIP profiling data and accounts for variable signal strength and positioning of significant patterns within profile regions by incorporating alignment and the option of signal normalisation in the profile comparison. CATCHprofiles supports the analysis workflow by an interactive graphical visualization of data and results.

Two other analysis tools are currently available that include aligning of ChIP profile regions. The first one, ChromaSIG [16], implements a heuristic clustering and alignment based on Gibbs sampling [17]. The second, ArchAlign [18], performs exhaustive alignment using a similar approach to the CATCH algorithm, but does not perform clustering. The non-exhaustive and probabilistic search of ChromaSIG has an advantage in speed, but also the disadvantage of varying, non-deterministic results. Also, the heuristic approach to alignment and clustering cannot guarantee sensitivity, and some patterns may go undetected. CATCHprofiles and ArchAlign circumvent this by performing an exhaustive comparison of all pairwise profile windows in the dataset. However, since ArchAlign does not perform clustering, but reports the average aligned pattern of a set of preselected profiles, it cannot be used for discovery of more than one pattern in the given data. Our CATCHprofiles tool presents advantages over both ChromaSIG and ArchAlign, since we include both hierarchical clustering and exhaustive alignment in a deterministic algorithm. Furthermore, the Java tool CATCHprofiles has an interactive graphical user interface to browse and export results and the CATCH core algorithm is implemented for parallel execution on multi-core machines.

CATCHprofiles can be used to detect ChIP profile patterns in an unbiased approach, i.e. not based on functional annotation, as well as to extract new biological information from the alignment of individual patterns. We demonstrate the power of CATCHprofiles by genome-wide clustering of H2A.Z-enriched sites in a ChIP-seq dataset, revealing the H2A.Z context to contain various patterns of CTCF, RNA Polymerase II (PolII) and histone modifications. We also show how the orientation of the individual ChIP profiling patterns correlates with the orientation of genomic elements, namely how the relative orientations of the H2A.Z and CTCF peak patterns are correlated with the orientation of the CTCF binding motif.

## Results

### The CATCH algorithm

We designed and implemented the CATCH algorithm to perform simultaneous alignment and clustering of ChIP profile patterns. To run the CATCH algorithm, the user must provide one or more ChIP profiling data sets along with the genomic regions to analyse, e.g. peak regions of interest. In the following, we use the shorthand ‘profiles’ refer to genomic regions of the ChIP profiling data, unless stated otherwise. Our implementation represents the profiles internally as multi-dimensional vectors of equidistant floating point values along their specified regions of the genome.

The CATCH algorithm uses a hierarchical clustering approach combined with pairwise alignment: it keeps a pool of profiles from which it iteratively aligns all pairs and chooses the most similar pair. Initially, this pool is the set of all profiles in the data set. Each time the most similar profile pair (P_1_, P_2_) is chosen, P_1_ and P_2_ are merged to obtain P′, the average profile of their alignment, and P_1_ and P_2_ are replaced by P′ in the profile pool. P′ is then aligned to all the remaining profiles in the pool to determine their pairwise similarity. The sequence of merging events determines the topology of the tree. Conceptually this type of clustering is an unweighted pair-group centroid clustering [19]. As default similarity measure for comparing the profiles we use the sum of squared distances and every profile pair is compared in both forward (left-to-right) and reverse (right-to-left, i.e. mirrored) direction. Each profile pair is aligned in the orientation (mirrored or non-mirrored) that gives the highest similarity. CATCH represents the profiles internally by a series of signals for fixed equidistant positions within the profile window, estimating missing values by linear interpolation of neighbouring signals, thereby allowing comparison of profiles with varying resolution. As profiles are aligned at different offsets, the generated average profile may grow in length. To avoid wasting computation time and introducing artefacts by aligning non-informative parts of the signal, the algorithm includes a measure for pruning signal at the edges of the alignment. The pruning in combination with the clustering is enforcing the idea of pattern significance by recurrence, since the most heavily aligned part of the pattern will be kept and the less densely aligned edges will be trimmed. The CATCH algorithm and the options for the similarity measure, normalization and pruning are described in detail in Supplementary Methods S1: CATCH algorithm and Figures S1, S2, S8, S9, S10, and Tables S1, S2. In the analysis of the H2A.Z profiles (see below), the default parameters were used.

### Visualization and graphical interface

CATCHprofiles is a stand-alone tool for ChIP profiling clustering analysis and visualization. The tool implements the CATCH algorithm, as described above, for the alignment and clustering of ChIP profiles. It takes as input selected areas from the ChIP profiling data, e.g. areas obtained from peak calling, or areas selected from annotation, such as promoter regions. Through the graphical user interface, the user can selectively load one or more ChIP profiling data sets, along with a bed format file defining the positions of the profiles to be analysed within the selected profiling data. When the data has been loaded into CATCHprofiles, the selected profiles are presented to the user in the Graph view with each included ChIP profiling experiment plotted in a different colour for easy distinction (Figure S4). After alignment and clustering, the result is visualized in two different types of displays, the Cluster view (Figure S5) to explore the tree obtained by the clustering, and the Branch view to visualize and compare profile patterns at selected branches of the tree. The graphical interface allows the user to examine and select distinctive ChIP-profile patterns and the corresponding branches of the tree for further analysis. At any level in the tree the average profile patterns and the genomic positions of the profiles can be exported as plain text while clusters can be marked and saved for later browsing in CATCHprofiles.

### Computational efficiency

The exhaustive all-against-all comparison and alignment in the CATCH algorithm comes at a cost in computation time. Since the similarity score is calculated per track in the pairwise comparisons, adding more ChIP profiling experiments (signal tracks) to the profiles adds linearly to the computation time. Adding more profiles, however, causes a quadratic increase in pair-wise profile comparisons and computation time. We have therefore implemented the CATCH clustering algorithm in C, optimizing for both memory efficiency and computation speed. Furthermore, we have enabled parallel computation of the comparison scores, so the computation time scales inversely with the number of available processors (see Supplementary Methods S1: Parallel Implementation).

### Clustering of PolII sites and alignment of promoters

**Figure 1:**
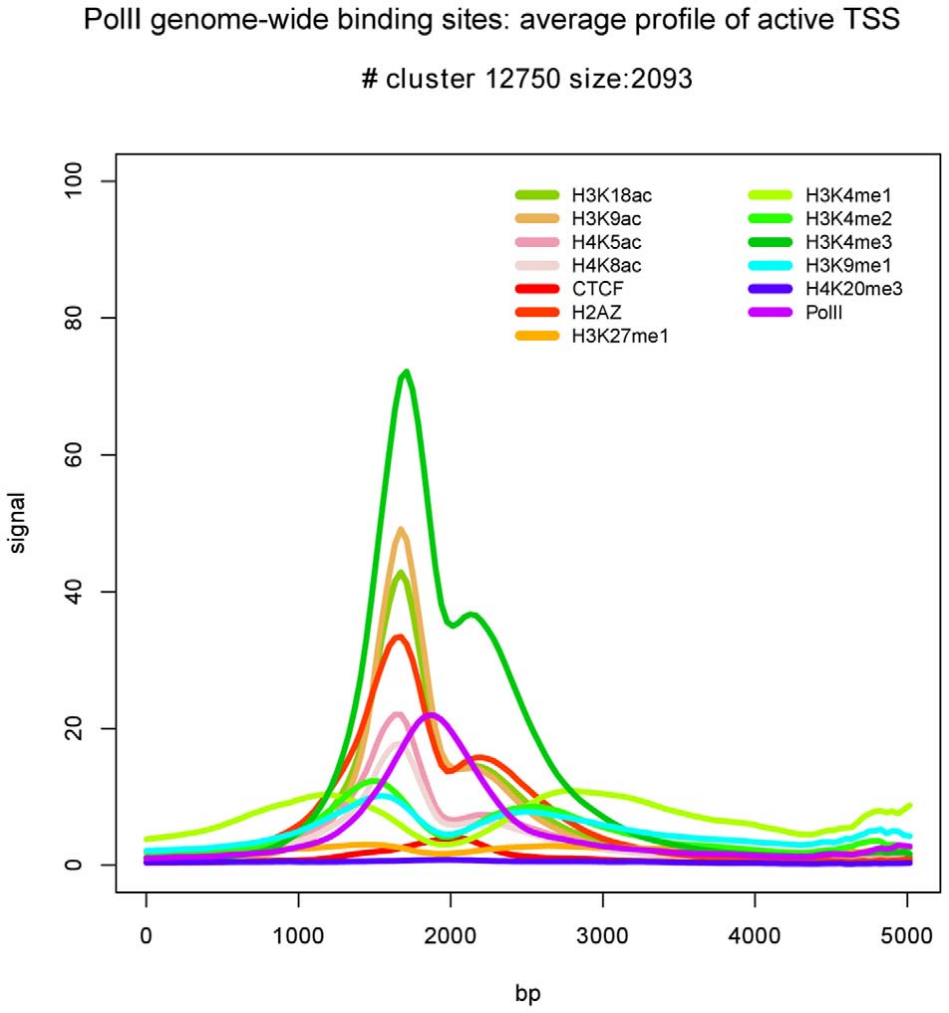
Example profile of PolII cluster with marks of active transcription. The average profile pattern of cluster 12750 (containing 2093 profiles) from the CATCH clustering of PolII binding sites. The profile pattern has a high signal for both H3K4me3 and all the histone acetylation marks, which are known to correlate with active transcription. 81% of the profiles are within 1 kb of annotated Ensembl TSS, and of the remaining 389 regions, 253 were within 1 kb of Aceview predicted TSS.

We demonstrate the capability of CATCH for unbiased discovery by clustering regions of PolII binding in the ChIP-seq dataset of PolII, H2A.Z and a selection of histone modifications from Wang *et al.* [15]. In these data CATCHprofiles detects a cluster of 2093 profiles with a high signal for H3K4me3 and for almost all the histone acetylation marks under study, a profile pattern that has been reported for actively transcribed promoters [15] (Figure 1). We validated the positions of the profiles in the cluster to be enriched in promoters by comparing to annotation. Indeed, 81% of the profiles are within 1 kb of annotated Ensembl TSS. From the remaining 19% more than half (253/389) were within 1 kb of TSS predicted by Aceview [20] based on transcription data (Supplementary material: cluster12750.xls).

**Figure 2:**
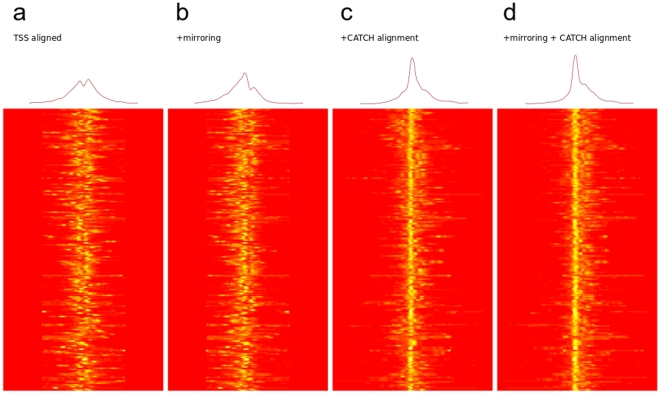
The effect of CATCH alignment on H3K4me3 profile on a subset of ENCODE TSS. A set of 241 promoter regions with high H3K4me3 was selected from the CATCH analysis of ENCODE TSS. The H3K4me3 signal is shown (a) aligned by the genomic position of the TSS disregarding the direction of the TSS (b) aligned by TSS and allowing mirroring of profiles to increase similarity of the patterns (c) by CATCH alignment without mirroring (d) by CATCH alignment and mirroring. The alignment becomes better and the average signal more localized when using both mirroring and CATCH alignment.

We used the same dataset to study how the alignment changes the average profile of the promoters. We selected the active promoters (TSS) from ENCODE regions and used CATCHprofiles to align the H3K4me3 signals in the promoter regions. When disregarding the direction of transcription, the average TSS has a peak of H3K4me3 on both sides of the centre (Figure 2A). However, the average profile patterns change when allowing both alignment and mirroring of the profile regions (Figure 2 B, C), revealing that the individual profile patterns are actually asymmetric around the TSS (Figure 2D).

### Clustering of H2A.Z profiles

To demonstrate the power of CATCH for the discovery of new, potentially biologically relevant patterns in ChIP-seq data we analysed the chromatin modification patterns accompanying H2A.Z. H2A.Z is a histone variant that is found throughout the genome. In both yeast and human, H2A.Z occupies two consecutive nucleosomes around the nucleosome-free region at transcriptionally active promoters [21], but little is known about binding patterns at other H2A.Z sites and their functional relevance.

**Figure 3:**
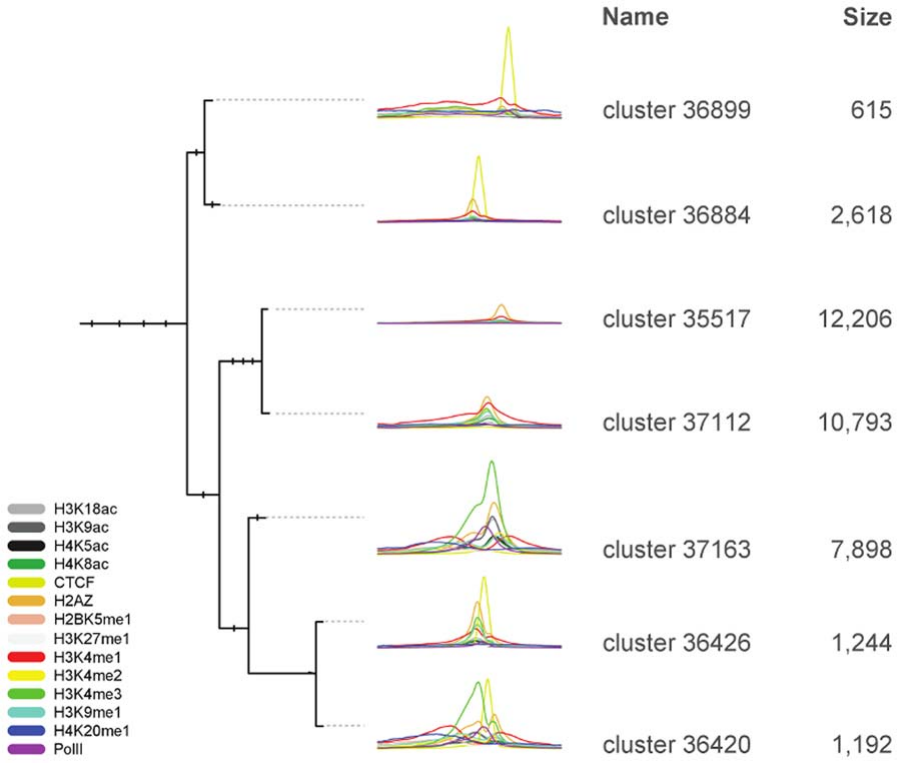
Dendrogram with overview of H2A.Z clusters. The tree of the 37456 H2A.Z profiles has been collapsed to show only the relation and patterns of the seven main clusters. Cluster profile patterns are shown in detail in Figure S6.

We applied the CATCH algorithm and the CATCHprofiles tool with our default settings to analyse the patterns around H2A.Z enriched sites in a genome-wide ChIP-seq dataset from human CD4+ cells of histone modifications, RNApolII and CTCF [15]. The dendrogram of the total 37456 ChIP-seq profile regions contained seven major clusters (Figure 3). Each of the clusters presented a unique combination and shape of binding patterns around the H2A.Z signal. The average profiles of the clusters were viewed and exported from the CATCHprofiles tool.

One cluster pattern (cluster 35517) consisted of H2A.Z binding sites with no apparent PolII, CTCF or histone mark. Another cluster (cluster 37112) has an H2A.Z peak co-located with peaks for H3K4me and H3K9me. Two of the clusters (cluster 37163 and 36420) have patterns closely resembling the known pattern of active promoters [6], [15], the main difference between them is that cluster 36420 has a CTCF peak immediately adjacent to the PolII peak while cluster 37163 has no CTCF. And finally, three clusters (cluster 36426, 36884 and 36899) have novel and asymmetric patterns with a CTCF peak flanking the H2A.Z and around them varying degrees of histone methylation (Figure 3 and Figure S6).

For each cluster, we extracted and compared the genomic context of the regions in the cluster with the whole-genome distribution of H2A.Z sites to asses which cluster pattern was over-represented in genomic regions located at 5′ end of genes, 3′ end of genes, in introns, in exons and gene distant regions (see Methods).

Gratifying, the two clusters that contain patterns resembling active promoters (cluster 37163 and 36420) contained regions close to annotated promoter regions (83% and 80% were within 5 kb of annotated TSS, respectively).

### CTCF/H2A.Z asymmetric patterns

Of particular interest are the three clusters in which the CTCF protein co-occurs with H2A.Z. Each of these three clusters is significantly over-represented in 3′ regions of genes as compared to the complete set of H2A.Z sites (Figure S7). These clusters show a pattern of H2A.Z located asymmetrically near the CTCF binding sites. Instead of an H2A.Z double peak as is seen in the promoter pattern, H2A.Z is present only on one side of the CTCF and thus incorporated in only one of the two neighbouring nucleosomes.

CTCF (CCCTC-binding factor) is a zinc finger protein that has been reported to be critical in regulation of gene expression [22]. The distinct positioning relative to the H2A.Z site uncovered by CATCHprofiles suggests a (possibly indirect) physical link between the CTCF binding site and the adjacent H2A.Z nucleosome. To corroborate the asymmetry of the CTCF/H2A.Z patterns we performed a CTCF motif detection for each profile region and correlated the motif orientation with the orientation of the profile in the CATCH alignment. The orientation of the CTCF/H2A.Z pattern has a highly significant correlation with the orientation of the CTCF motif for each of the clusters that feature the CTCF/H2A.Z peak pattern: cluster 36884 (0.33, P<e-32), cluster 36426 (0.39, P<e-19 ), cluster 36420 (0.29, P<e-5 ) while there was no correlation in the remaining clusters (Table 1).

**Table 1:** Correlation of pattern orientation with orientation of CTCF motif for each of the H2A.Z clusters.

Cluster name	Brief description	Cluster size	CTCF Motif correlation
36899	Low H2A.Z+CTCF+H3K4me1	615	0.040
36884	H2A.Z+CTCF	2,618	0.326
35517	H2A.Z alone	12,206	0.098
37112	H2A.Z+met	10,793	0.075
37163	H2A.Z+Promoter	7,898	0.019
36426	H2A.Z+CTCF+met	1,244	0.390
36420	H2A.Z+Promoter+CTCF	1,192	0.285

Only the CTCF containing patterns with a clear H2A.Z peak show correlation with the orientation of the CTCF motif. Promoter: Marks of active promoters including PolII, histone acetylation and histone methylation marks. Met: Histone methylation.

**Figure 4:**
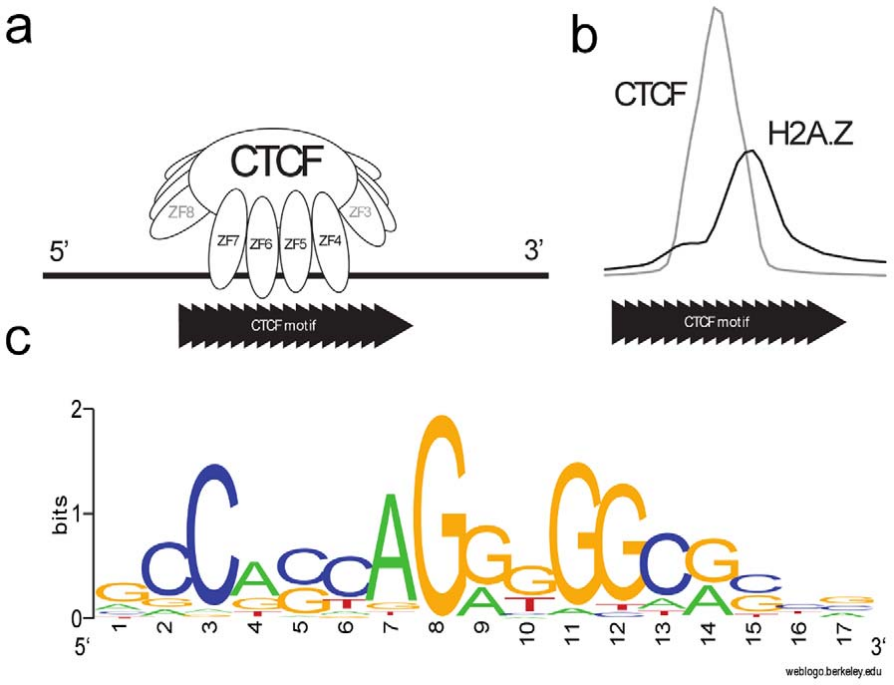
The orientation of the CTCF/H2A.Z pattern is correlated with the orientation of the CTCF binding motif. (a) Of the eleven zincfingers in CTCF, only four are required for strong binding. The orientation of the binding with respect to the CTCF motif was determined by Renda et al [23]. (b) The dominant orientation of the CTCF/H2A.Z pattern with respect to the orientation of the underlying CTCF motif. (c) The CTCF motif as derived from motif detection in genome-wide CTCF peaks in the ChIP-seq dataset of Barski et al [6].

The CTCF binding affinity to the CTCF motif was investigated by Renda *et al* [23] who showed that of the eleven zinc fingers in the protein, only four are required for strong binding, and these zinc fingers (numbered ZF4 to ZF7) have a specific orientation with respect to the sequence motif. The correlation of the asymmetric CTCF/H2A.Z pattern with the CTCF binding motif indicates that the H2A.Z nucleosome is most likely to be found 3′ of the CTCF motif that corresponds to the ZF4 side of the bound CTCF protein (Figure 4).

Two earlier studies on CTCF and nucleosome positioning that did not apply alignment did not report any asymmetric patterns, but instead showed that H2A.Z is highly enriched in nucleosomes flanking the CTCF binding sites [24], and that H2A.Z has one major enrichment peak at the centre of intergenic CTCF-sites [25]. In their recent paper, Lai and Buck [18] did report an asymmetry in the nucleosome pattern as well as in the H2A.Z pattern when they aligned the signal in both forward and reverse direction around a preselected set of 1000 CTCF binding sites. However, in their study Lai and Buck did not find a correlation between the pattern orientation and the orientation of the underlying CTCF motif that links the asymmetry of the pattern to the orientation of the CTCF protein.

## Discussion

The analysis of ChIP profiling data aims to discover the functional relevance of DNA-binding proteins. A prerequisite for such discovery is to be able to either detect patterns in sites of known functionality, or the opposite, to interrogate and annotate the function of sites with specific patterns. Both of these approaches require a method for clustering the ChIP profile patterns, and for this purpose we developed CATCHprofiles - a ChIP profile clustering and alignment algorithm integrated in a Java tool to visualize and browse the results.

We designed the CATCH algorithm specifically to handle the structure of ChIP profiling data, including taking advantage of the genome-wide coverage for unbiased discovery: Firstly, CATCH performs an exhaustive comparison and clustering based solely on the signal patterns in the profiles, thus eliminating the need to incorporate pre-existing knowledge, like the presence of Transcription Start Sites, into the search for patterns. Secondly, because the CATCH clustering includes alignment of the profiles, we do not need e.g. annotated Transcription Start Sites (TSS) to align the promoters, and we can actually improve the resolution of annotation-based profiles. When comparing, for known promoters, the average profile based on a TSS alignment with one based on a Chip-profile based alignment using CATCH, the resolution of the average profile improved markedly after CATCH alignment (Figure 2). Thirdly, because CATCH by default compares the profile regions by both their forward and reverse (mirrored) orientation during the alignment procedure, we can detect asymmetric patterns even if we have no prior knowledge about their direction, as shown for both promoters (Figure 2) and H2A.Z patterns (Figure 3). Fourthly, since the ChIP profiling signal can vary between experiments depending on e.g. the difference in affinity of the various antibodies, CATCH incorporates options for normalizing the signal between the experiments included in the clustering to prevent the dominance of e.g. a single high signal track (Figure S3). Finally, Chip profiling data can have various resolutions and coverage and the internal interpolation in CATCHprofiles allows seamless combination of data of various resolution and coverage.

Next to discovering and characterizing individual binding patterns, CATCH may also be applied to compare binding patterns between cell types. Or, within one cell type, to compare temporal variation in binding patterns by combining the ChIP profiling experiments from different time points. It should thereby be noted that the CATCH algorithm is not limited to ChIP profiling data, but can just as easily be applied to e.g. DNA methylation or DamID [26] profiles. In fact, CATCHprofiles is not dependent on the platform used to produce the data, and the pattern analysis can be applied for any genomic data where shape and the relative genomic location of the signals adds to the biological interpretation of the result.

The challenge for many high-throughput analysis techniques is the handling and visualization of the high-dimensional data. Often a viable solution is abstraction, as when plotting in the space of principal components when using principal component analysis [27] for clustering. But in the cases where representative and intuitive visualization is feasible, the tools that provide a graphical visualization often achieve the highest resonance in the scientific community, as was the case with the alignment program ClustalW which has had a full graphical interface since 1997 [28]. CATCHprofiles provides the ChIP profiling community with an efficient implementation of an exhaustive alignment and clustering algorithm alongside an easy-to-use interactive graphical display of the results.

CATCHprofiles - with example datasets and installation instructions - is available for download from http://catch.cmbi.ru.nl.

## Methods

### Implementation

The CATCHprofiles tool is implemented using a combination of two programming languages; Java and C. The graphical user interface is implemented in Java, while the CATCH clustering and alignment algorithm is implemented in C as the CATCHprofiles clustering engine. To accommodate the computational load of large-scale analysis we have optimized the CATCHprofiles clustering engine for parallel efficiency and achieved a close-to-linear, inverse scaling with the number of cores (Figure S9). The speed-up plot was produced from benchmarks on an 8-core system. Based on the algorithm design and the parallel implementation, the running time of the CATCHprofiles clustering engine scales quadratically with the number of profiles and linearly with the number of signal tracks. A more detailed description of the parallel implementation is available in Supplementary Methods: Parallel implementation.

### H2A.Z enriched sites

The binding sites were defined by peak calling on the H2A.Z ChIP-seq data from Wang et al [15] using the peak calling program MACS with default settings resulting in a total of 37456 sites. We then defined the profiles for the analysis as the 5000 bp windows around the H2A.Z sites and we selected 11 ChIP-seq tracks of histone modifications (H3K18ac, H3K9ac, H4K5ac, H4K8ac,H2BK5me1, H3K27me1,H3K4me1, H3K4me2, H3K4me3, H3K9me1, H4K20me1) together with H2A.Z, CTCF and PolII as input to the CATCH algorithm. The computation was executed in parallel on a 64-core machine. Determination of genomic context and the comparison of genomic distributions were done using the online tool PinkThing based on Ensembl NCBI 36 gene annotation (http://pinkthing.cmbi.ru.nl).

## Supporting Information

Figure S1 **The alignment of two signal sequences S_A_ and S_B_ is characterised by an integer r denoting the shift of sequence S_B_.** If r is positive, S_B_ is shifted r positions to the left, relative to S_A_. If r is negative, S_B_ is shifted -r positions to the right as shown in this figure. As a function of r, n_overlap_ is the length of the sequence overlap and n_total_ is the total length of the alignment.
(PNG)

Figure S2 **Conceptual illustration of the CATCH clustering algorithm.** Example of clustering four profiles with two tracks of ChIP profiling data, plotted in red and blue respectively. All pairs of profiles are aligned to find the alignment of highest similarity. In each iteration, the profile pair of highest similarity is clustered and their cluster is represented by their average aligned profile. The hierarchical clustering continues until all profiles and clusters are included in the dendrogram.
(PDF)

Figure S3 **Normalization affects the clustering and the resolution of the patterns.** (a) with normalization of the signal strength the profiles cluster by the intensity and shape of all tracks equally, resulting in a clear split between patterns of active and inactive promoters as highlighted in the dendrogram with green and red respectively. The inactive promoters pattern is low signal for all the tracks shown. Within the cluster I of active promoters subclusters arise with variations of the active promoter pattern, e.g. cluster II. (b) Without the use of normalization, the intensity of the signals dominates the clustering. Most of the inactive promoter patterns of low signal intensity are still clustered together, highlighted in red. However, the biggest cluster with a pattern resembling the active promoter pattern is cluster I, and it is clustered separately from e.g. cluster II which differs mainly in signal intensity. Clustering using normalization is the recommended and default option for clustering in CATCH to avoid the dominance of high signal tracks in the clustering.
(PNG)

Figure S4 **CATCH Graph view.** After loading a data set of ChIP profiles, the Graph view shows plots of all profile regions. On the left the track names and colours can be adjusted for easy distinction.
(PNG)

Figure S5 **Screenshot CATCH cluster view.** The result of the CATCH clustering algorithm is shown on the right as a dendrogram. The tree can be interactively browsed to examine the average profile patterns at any level in the tree. Individual profiles and subclusters can be exported by right-clicking on the cluster node in the tree. Below the tree, the average profile is shown for the currently selected cluster.
(PNG)

Figure S6 **Detailed view of the H2A.Z genome-wide cluster patterns.** Each pattern represents the average profile pattern for the profiles in the cluster. The patterns of clusters 36420 and 37163 contain high signals for PolII, methylation and acetylation marks correlating with active transcription. Four clusters (36420, 36426, 36884 and 36899) have a CTCF peak close to the H2A.Z. The genomic distributions corresponding to these clusters are shown in Figure S7.
(PDF)

Figure S7 **Genomic distributions of the seven clusters of H2A.Z binding sites.** Each plot shows the distribution of the categories: exon, intron, 5′near, 5′far, 3′near, 3′far and distant. The limit for ‘near’ regions is 5 kb, the limit for ‘far’ regions is 25 kb. The categories are shown as numbers relative to the H2A.Z genomic distribution with p-values indicating significant differences per category. The clusters with CTCF, but no acetylation marks, e.g. clusters 36426, 36884 and 36899, are all significantly enriched in the 3′ regions of genes.
(PDF)

Figure S8 **CATCH algorithm flow diagram indicating concurrent computation.** Score computation: the initial comparison and similarity score computation for all profile pairs. Find highest score: the selection of the highest scoring profile pair. Merge i and j having the highest score: the merging of the selected pair into a representative profile. Dependencies are visualized by arrows and parallel parts marked with the order of concurrency available.
(PNG)

Figure S9 **Speedup plot of the relative performance increase in the CATCHprofiles clustering engine.** The parallel implementation of the CATCH clustering engine results in a near-linear speedup of computation time with increased number of threads. The y-axis shows the speedup, and the x-axis the number of threads used. The profiles contain 8 tracks and the alignment was set to use a minimum overlap of 50%, the other parameters were set to default as listed in Error: Reference source not found.
(PNG)

Figure S10 **Running time dependence on alignment.** Running time of clustering 1480 profiles with 8 tracks, when the minimum overlap is varied. Results are shown for executions with 1, 4 and 8 threads.
(PDF)

Table S1 **CATCH algorithm options as described in Supplementary Methods.** Options indicated with an asterisk are the default selected options.
(DOC)

Table S2 **Time spent in the different parts of the CATCH algorithm as measured on three benchmark data sets.**
(DOC)

Supplementary Methods S1 **Detailed description of: The CATCH algorithm, profile similarity measures, signal normalization, representative profile of a cluster and parallel implementation.**
(DOC)
