# Supplementary figures and images for: CATCHprofiles: Clustering and Alignment Tool for ChIP Profiles
Source: PLoS One. 2012 Jan 4;7(1):e28272. doi: 10.1371/journal.pone.0028272 (PMC3251562; doi:10.1371/journal.pone.0028272)

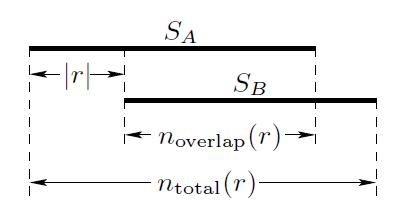

Supplement: Figure S1 — The alignment of two signal sequences SA and SB is characterised by an integer r denoting the shift of sequence SB. If r is positive, SB is shifted r positions to the left, relative to SA. If r is negative, SB is shifted -r positions to the right as shown in this figure. As a function of r, noverlap is the length of the sequence overlap and ntotal is the total length of the alignment. (PNG) [file pone.0028272.s001.png]

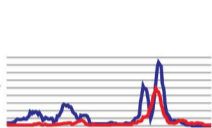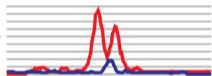

average profile pattern

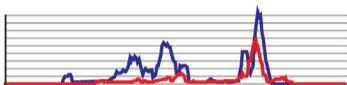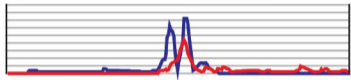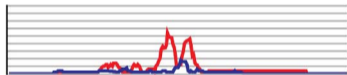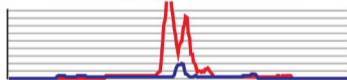

aligned profiles

Supplement: Figure S2 — Conceptual illustration of the CATCH clustering algorithm. Example of clustering four profiles with two tracks of ChIP profiling data, plotted in red and blue respectively. All pairs of profiles are aligned to find the alignment of highest similarity. In each iteration, the profile pair of highest similarity is clustered and their cluster is represented by their average aligned profile. The hierarchical clustering continues until all profiles and clusters are included in the dendrogram. (PDF) [file pone.0028272.s002.pdf]

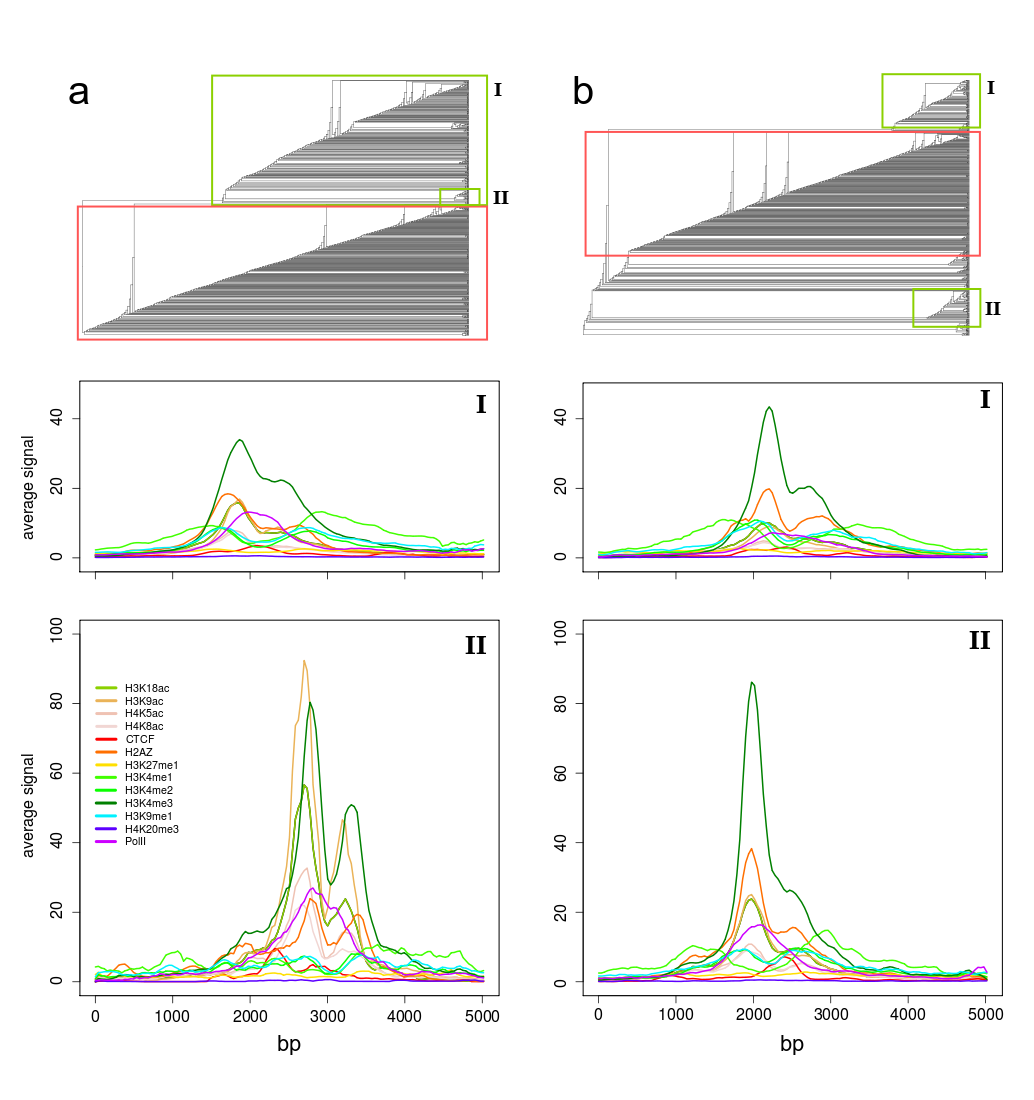

Supplement: Figure S3 — Normalization affects the clustering and the resolution of the patterns. (a) with normalization of the signal strength the profiles cluster by the intensity and shape of all tracks equally, resulting in a clear split between patterns of active and inactive promoters as highlighted in the dendrogram with green and red respectively. The inactive promoters pattern is low signal for all the tracks shown. Within the cluster I of active promoters subclusters arise with variations of the active promoter pattern, e.g. cluster II. (b) Without the use of normalization, the intensity of the signals dominates the clustering. Most of the inactive promoter patterns of low signal intensity are still clustered together, highlighted in red. However, the biggest cluster with a pattern resembling the active promoter pattern is cluster I, and it is clustered separately from e.g. cluster II which differs mainly in signal intensity. Clustering using normalization is the recommended and default option for clustering in CATCH to avoid the dominance of high signal tracks in the clustering. (PNG) [file pone.0028272.s003.png]

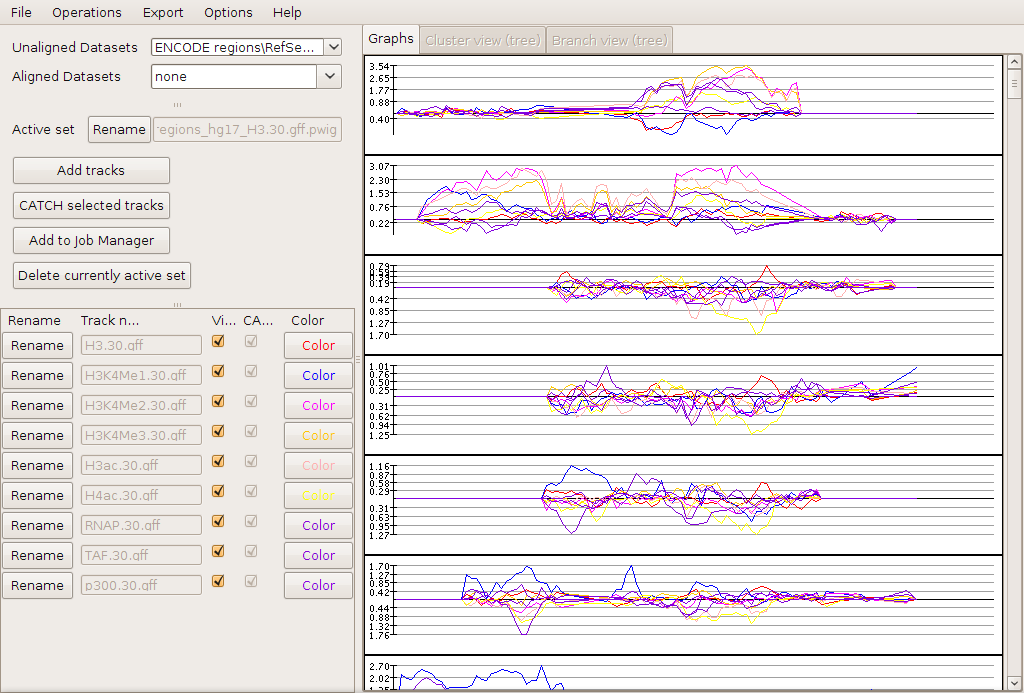

Supplement: Figure S4 — CATCH Graph view. After loading a data set of ChIP profiles, the Graph view shows plots of all profile regions. On the left the track names and colours can be adjusted for easy distinction. (PNG) [file pone.0028272.s004.png]

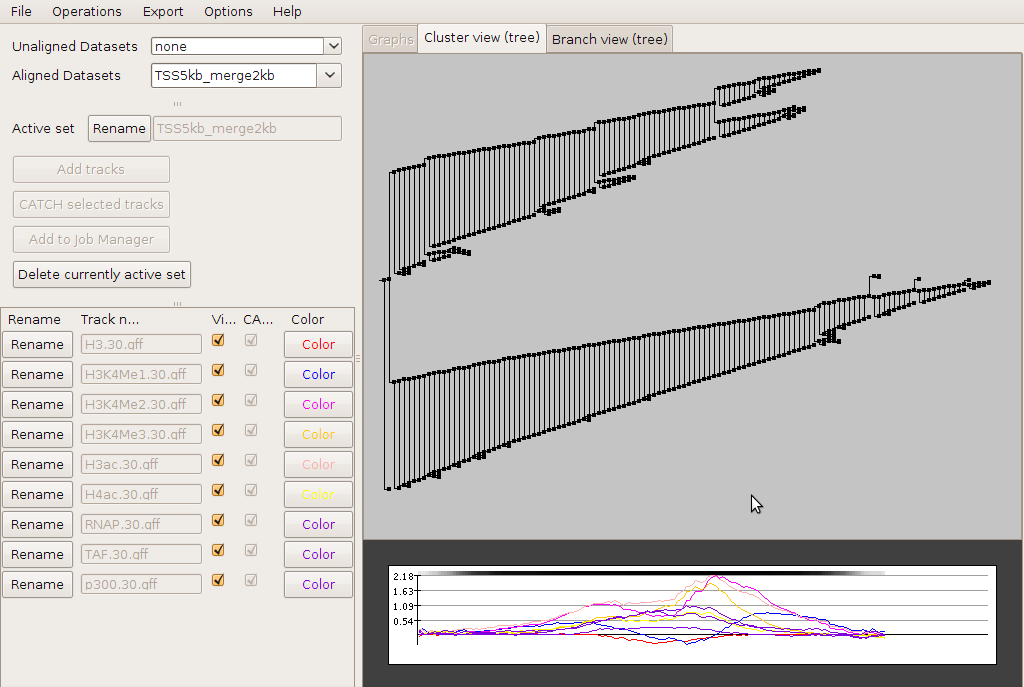

Supplement: Figure S5 — Screenshot CATCH cluster view. The result of the CATCH clustering algorithm is shown on the right as a dendrogram. The tree can be interactively browsed to examine the average profile patterns at any level in the tree. Individual profiles and subclusters can be exported by right-clicking on the cluster node in the tree. Below the tree, the average profile is shown for the currently selected cluster. (PNG) [file pone.0028272.s005.png]

cluster36420

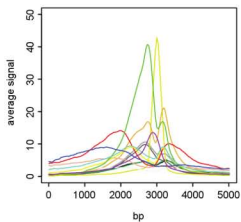

cluster36426

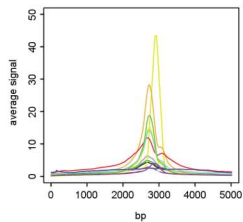

cluster37163

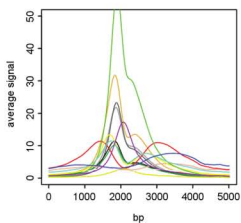

cluster37112

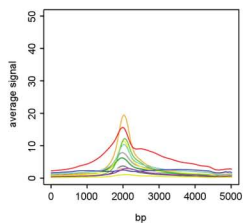

cluster35517

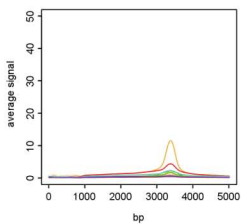

cluster36884

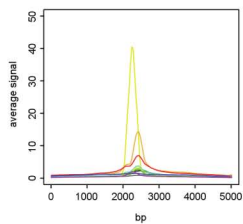

cluster36899

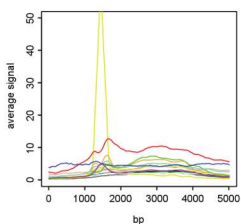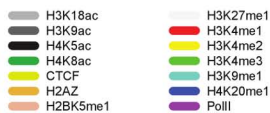

Supplement: Figure S6 — Detailed view of the H2A.Z genome-wide cluster patterns. Each pattern represents the average profile pattern for the profiles in the cluster. The patterns of clusters 36420 and 37163 contain high signals for PolII, methylation and acetylation marks correlating with active transcription. Four clusters (36420, 36426, 36884 and 36899) have a CTCF peak close to the H2A.Z. The genomic distributions corresponding to these clusters are shown in Figure S7. (PDF) [file pone.0028272.s006.pdf]

cluster36420

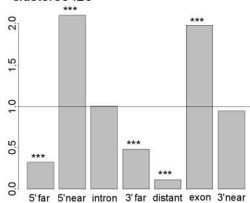

cluster36426

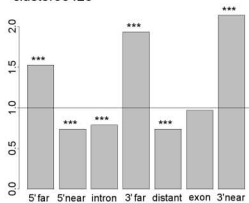

cluster37163

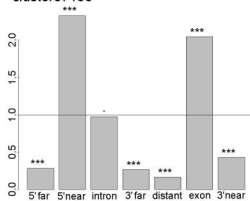

cluster37112

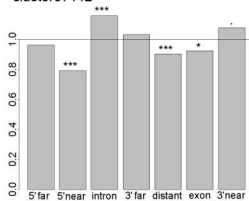

cluster35517

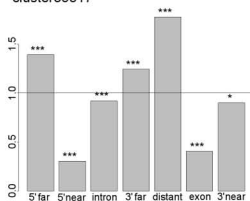

cluster36884

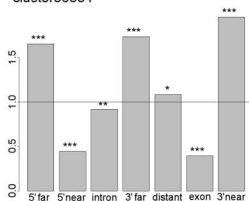

cluster36899

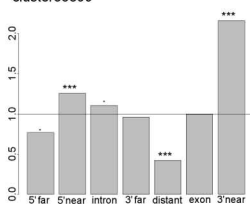

Supplement: Figure S7 — Genomic distributions of the seven clusters of H2A.Z binding sites. Each plot shows the distribution of the categories: exon, intron, 5′near, 5′far, 3′near, 3′far and distant. The limit for ‘near’ regions is 5 kb, the limit for ‘far’ regions is 25 kb. The categories are shown as numbers relative to the H2A.Z genomic distribution with p-values indicating significant differences per category. The clusters with CTCF, but no acetylation marks, e.g. clusters 36426, 36884 and 36899, are all significantly enriched in the 3′ regions of genes. (PDF) [file pone.0028272.s007.pdf]

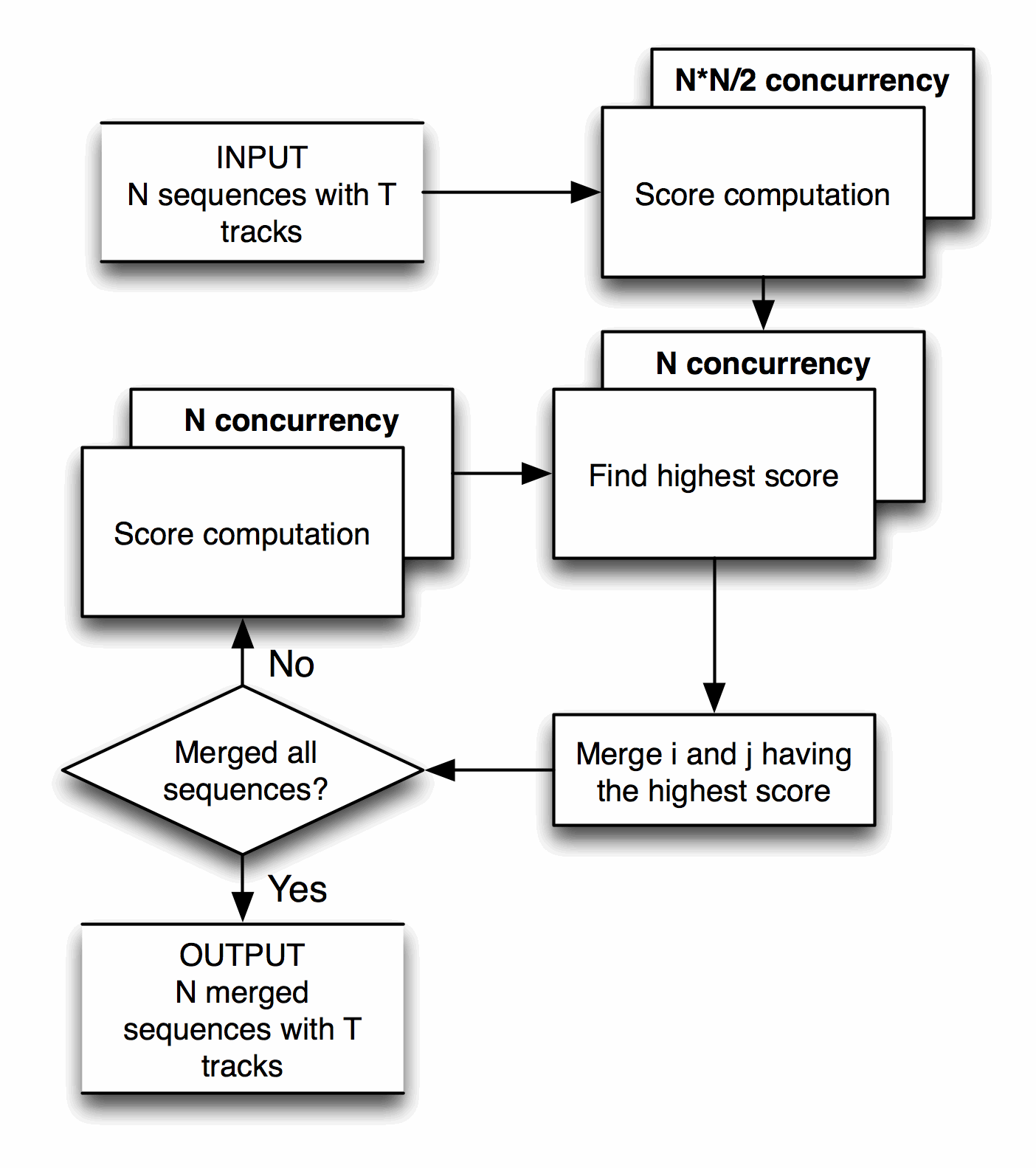

Supplement: Figure S8 — CATCH algorithm flow diagram indicating concurrent computation. Score computation: the initial comparison and similarity score computation for all profile pairs. Find highest score: the selection of the highest scoring profile pair. Merge i and j having the highest score: the merging of the selected pair into a representative profile. Dependencies are visualized by arrows and parallel parts marked with the order of concurrency available. (PNG) [file pone.0028272.s008.png]

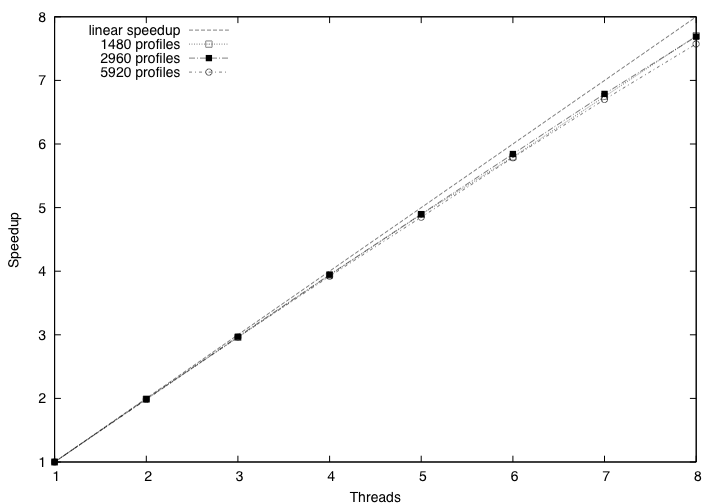

Supplement: Figure S9 — Speedup plot of the relative performance increase in the CATCHprofiles clustering engine. The parallel implementation of the CATCH clustering engine results in a near-linear speedup of computation time with increased number of threads. The y-axis shows the speedup, and the x-axis the number of threads used. The profiles contain 8 tracks and the alignment was set to use a minimum overlap of 50%, the other parameters were set to default as listed in Error: Reference source not found. (PNG) [file pone.0028272.s009.png]

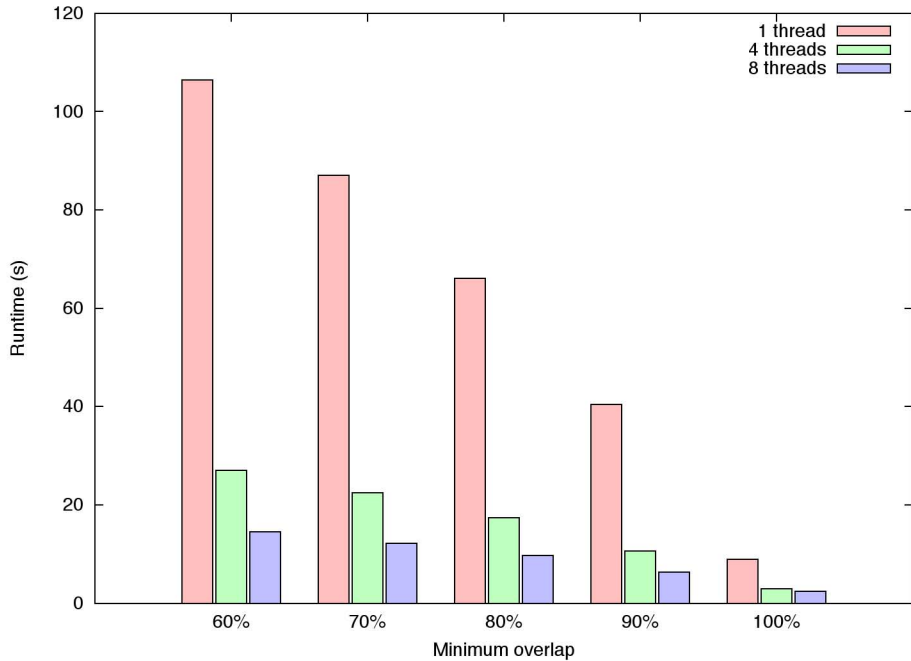

Supplement: Figure S10 — Running time dependence on alignment. Running time of clustering 1480 profiles with 8 tracks, when the minimum overlap is varied. Results are shown for executions with 1, 4 and 8 threads. (PDF) [file pone.0028272.s010.pdf]
