# Supplementary material for: CATCHprofiles: Clustering and Alignment Tool for ChIP Profiles
Source: PLoS One. 2012 Jan 4;7(1):e28272. doi: 10.1371/journal.pone.0028272 (PMC3251562; doi:10.1371/journal.pone.0028272)
Supplement: Table S1 — CATCH algorithm options as described in Supplementary Methods. Options indicated with an asterisk are the default selected options. (DOC) [file pone.0028272.s011.doc]

| **Parameter** | **Default** | **Options** |
| --- | --- | --- |
| Weighted merge | * | Yes |
|  |  | No |
| Similarity Score |  | CC |
|  | * | SSD |
|  |  | weighted SSD |
|  |  | Pearson |
| Normalization |  | None |
|  |  | Sum of both |
|  | * | Largest maximum value |
|  |  | Largest average value |
| Maximum pruning | 1/15 | (percentage) |
| Minimum overlap | 1/5 | (percentage) |

Table S1: CATCH algorithm options as described in Supplementary Methods.Options indicated with an asterisk are the default selected options.
