# Supplementary material for: CATCHprofiles: Clustering and Alignment Tool for ChIP Profiles
Source: PLoS One. 2012 Jan 4;7(1):e28272. doi: 10.1371/journal.pone.0028272 (PMC3251562; doi:10.1371/journal.pone.0028272)
Supplement: Table S2 — Time spent in the different parts of the CATCH algorithm as measured on three benchmark data sets. (DOC) [file pone.0028272.s012.doc]

| **Profiling benchmark**  **data set** | **Initial similarity score computation** | **Selecting the highest scoring profile pair** | **Updating similarity scores after merging** | **Merging profiles** | **Other** |
| --- | --- | --- | --- | --- | --- |
| 1480 profiles, 1 track | 40.71% (27.6s) | 4.51% (3.1s) | 53.94% (36.5s) | 0.75% (0.5s) | 0.09% (0.1s) |
| 1480 profiles, 2 tracks | 42.19% (53.8s) | 2.37% (3.0s) | 55.15% (70.3s) | 0.23% (0.3s) | 0.06% (0.1s) |
| 2960 profiles, 1 track | 43.80% (110.0s) | 9.11% (22.9s) | 46.85% (117.7s) | 0.19% (0.5s) | 0.06% (0.1s) |

Table S2: Time spent in the different parts of the CATCH algorithm as measured on three benchmark data sets.
