# Supplementary material for: CATCHprofiles: Clustering and Alignment Tool for ChIP Profiles
Source: PLoS One. 2012 Jan 4;7(1):e28272. doi: 10.1371/journal.pone.0028272 (PMC3251562; doi:10.1371/journal.pone.0028272)
Supplement: Supplementary Methods S1 — Detailed description of: The CATCH algorithm, profile similarity measures, signal normalization, representative profile of a cluster and parallel implementation. (DOC) [file pone.0028272.s013.doc]

Supplementary Methods

The CATCH algorithm

The CATCH algorithm uses a standard hierarchical clustering approach: it keeps a pool of profiles from which it iteratively chooses the most similar pair, according to the chosen similarity measure. Initially, this pool is the set of input profiles. Each time a most similar pair $(P_{1},P_{2})$ is chosen, $P_{1}$ and $P_{2}$ are merged to a representative profile $P^{'}$, and $P_{1}$ and $P_{2}$ are replaced by $P^{'}$ in the profile pool. The sequence of merging determines the topology of the dendrogram (Figure S2).

When running CATCHprofiles, it is possible to choose between different similarity scores, normalization methods, etc. These options are described in the following sections and summarized in Table S1.

Profile similarity measures

The choice of the alignment scoring measure will determine which type of signal similarity is given the highest score and thus also determine the sequence of clustering of the profiles. CATCH implements three different scoring schemes, described in detail in the sections below:

- *Cross Correlation* (CC), which is used extensively in signal analysis, see e.g. Smith[1]⁠. It is equivalent to the vector dot product, which is also sometimes called the inner product.
- *Pearson's Correlation Coefficient* (PC), which measures the linear correlation between two signals. It can be seen as a normalized version of CC.
- *Sum of Squared Differences* (SSD).

The three scoring measures are all described in Gelder et al[2]⁠.

For two profiles containing multiple tracks of signal sequences, the scores are calculated per track, and the alignment score for the complete profile is computed as the average of the alignment score per track.

The alignment of two signal sequences $S_{A}[0..n_{A}-1]$ and $S_{B}[0..n_{B}-1]$ is characterised by an integer $r$. If $r$ is positive, $S_{B}$ is shifted $r$ positions to the left, relative to $S_{A}$. If $r$ is negative, $S_{B}$ is shifted $-r$ positions to the right. The latter situation is illustrated in Figure S1.

For a given score, CC, PC, or SSD, the  *alignment score* is calculated for each shift , where and $p\in[\text{0;1}]$ is a parameter that can be adjusted. The  *similarity score* of $S_{A}$ and $S_{B}$ is the alignment score of the best shift. Thus, if high values correspond to a good score, like PC and CC, the similarity score is calculated as

and if low values correspond to a good score, like SSD, the similarity score is calculated as

The following notation is used in the descriptions below (See Figure S1 for more intuitive definitions).

For each shift $r$, let $n_{\text{overlap}}(r)$ denote the number of positions in the alignment where both sequences are defined. Then,

Similarly, let $n_{\text{total}}(r)$ denote the total number of positions in the alignment, i.e.

Finally, let

and

Cross Correlation

The cross correlation alignment score is defined for sequences $S_{A}$ and $S_{B}$ as the sum of the signal products at all positions in the alignment where both sequences are defined:

Large CC values correspond to good alignments, and low values correspond to bad alignments. The intuition is: If two similar signals are aligned correctly, high signal values are aligned with high signal values, and thus, the high values are amplified as much as possible. An advantage of the cross correlation score is that it is very noise insensitive.

Pearson's Correlation Coefficient

Pearson's correlation coefficient can be seen as a variant of the cross correlation coefficient, where the signals are first normalized by subtracting the mean and dividing by the standard deviation times the square root of the length of the signal. It measures linear correlation and is defined as

where

is the average value of $S_{A}$ in the overlap with $S_{B}$ and

is times the standard deviation of $S_{A}$ in the overlap with $S_{B}$. Similarly,

is the average value of $S_{B}$ in the overlap with $S_{A}$ and

is times the standard deviation of $S_{B}$ in the overlap with $S_{A}$.

The possible values of Pearson's Correlation Coefficient lie between $-1$ and $1$. A value of $1$ means that the two signals are perfectly linearly correlated, a value of $0$ means that there is no linear relationship between the signals, and a value of $-1$ means that they are anti-correlated.

Sum of Squared Differences

When using the sum of squared differences as the scoring measure, one could decide to consider only positions where both sequences are defined, as with the CC score:

However, this would generally favour alignments with very large shifts, since they would result in very few terms in the sum.

One solution to this could be to weight the sum by the ratio of the total number of positions in the alignment to the number of overlap positions:

This is called weighted SSD.

Another solution could be to consider the full alignment and represent missing signal values by 0:

where $S_{A}[i]\text{=0}$, for $i\text{<0}$ or $i\geq n_{A}$, and $S_{B}[i]\text{=0}$, for $i\text{<0}$ or $i\geq n_{B}$. Thus, shifting high signal values of one signal past one end of the other signal is penalized significantly more than having small signal values sticking out. CATCH implements both options.

Signal normalization

Depending on the choice of similarity measure, high signal values may be either favoured (CC) or disfavoured (SSD) in the calculation of the similarity score. By normalizing the profiles before calculating the alignment and similarity scores, the dependence on signal strength can be removed or weakened. It may be desirable to let the normalization factor depend on both signals. Thus, when comparing two signals $S_{A}$ and $S_{B}$, each signal value in $S_{A}$ is divided by a normalization factor $\text{Norm}(S_{A},S_{B})$ depending on $S_{A}$ and possibly on $S_{B}$. Similarly, each signal value in $S_{B}$ is divided by a normalization factor $\text{Norm}(S_{B},S_{A})$ depending on $S_{B}$ and possibly on $S_{A}$.

With Pearson's Correlation Coefficient, additional normalization is not necessary, since the normalization is “built-in” in the measure. The built-in normalization ensures that the results lie between $-1$ and $1$ and thus, in a sense, it improves the interpretation of the results. On the other hand, we do not have the flexibility of choosing between different normalizations. Thus, e.g. two signals that are identical except for a scaling factor will score the same as two signals that are exactly identical.

Sum of Values

Normalizing by the sum of values ensures that only the shape of the signal is evaluated in the similarity measure. However, this also entails that comparing two signal shapes $S_{A}$ and $S_{B}$ that are the same except for a scaling factor, will have the same similarity as comparing $S_{A}$ with itself.

Largest Maximum Value

If it is desirable to take the scaling into account, so two identical signals will score higher than the same two signals differing by a scaling factor, it is an option to normalize by the maximum value of either sequence. Thus,

Largest Average Value

As a second way of taking the scaling into account, CATCH also implements normalization by the maximum average signal value of $S_{A}$ and $S_{B}$.

Representative profile of a cluster

Every time a pair of profiles is selected as the pair with the highest similarity score, the two profiles are merged to a new representative profile for the cluster. Each position in the representative profile is the (weighted) average of the corresponding positions in the two merged profiles. The merging of profiles has to take into account that the aligned pair may have positions where only one of the two profiles have defined values, possibly due to the shift of the alignment. The merging may also account for the number of original profiles represented by each of the two profiles.

Weighted merging

When merging two profiles, the number of original profiles represented by each of the two profiles may differ.

If weighted merging is chosen, the new representative profile will be a weighted average of the two merged profiles, where the weights are defined per position by the total number of original profiles in each of the two merged profiles.

Pruning (trimming) the alignments

To determine the merged profile even when there are positions with undefined values for one of the two profiles in the alignment, there are two main options:

1. Cut away the positions with undefined values, so the merged profile will contain only positions with defined values for both profiles
2. Use the value of the profile that does have a value defined at that position

CATCH will prune merged profiles from left and right until reaching a position where the weight is above a given threshold defined as a percentage of the maximum weight of the alignment. The threshold can be set as a parameter (Table S1). The second case above corresponds to a threshold of 0. The default threshold weight for pruning is 1/15, i.e. pruning parts of the alignment where less than one out of fifteen merged profiles align.

Options

A number of options and parameters can be set for adjusting the alignment and clustering. Available algorithm options are listed in Table S1.

Parallel implementation

When activating a clustering of N selected profiles from the CATCHprofiles java application, the entire job description of data and user-selected parameters are compiled in JSON format and used as input to the CATCH engine written in C. The complete clustering result is then loaded into the java application for visualization.

The CATCHprofiles clustering algorithm has four main components: the initial comparison and similarity score computation for all profile pairs, the selection of the highest scoring profile pair, the merging of the selected pair into a representative profile, and the updating of the similarity score table (Figure S8: The clustering algorithm flow diagram). In Table S2 we show the time spent in each of these four main components for three different problem sizes. Increasing the number of tracks, requires more time for similarity score computation while reducing the percentage of time spent selecting the highest scoring profile pair. Increasing the number of profiles adds to the percentage of time spent selecting the highest scoring profile pair. Since both variations are equally important we must optimize for both cases.
We have written a threaded (parallel) implementation of both the pairwise score computations and the selection of the highest scoring profile pair. Since all the parts were contained in loops and we wanted the threaded code to be platform independent, the OpenMP (Open Multi-Processing) API was used to implement the threading. It handles creating and terminating threads, synchronization between threads, shared and local variables and dividing the workload between the created threads.

The running time of the CATCHprofiles clustering engine depends on the size of the input data, the user-specified parameters (see Table S1) and the available hardware. We benchmark the CATCHprofiles clustering engine using a test data set of 1480 profiles, 2960 profiles and 5920 profiles, all with 8 tracks of 52 data points per profile. The test data sets of 2960 and 5920 profiles were generated by replicating the data set of 1480 profiles. The size of the data set varies with the number of profiles and tracks. The benchmarks are performed by executing the clustering engine in isolation, thus communication with the Java application is not included in the measurements.

The CATCH executable outputs the processing time spent clustering profiles. For specifying the amount of threads, the environment variable OMP_NUM_THREADS is set to the desired threadcount. If OMP_NUM_THREADS is not set, then OpenMP automatically uses a threadcount that matches the available cores on a system. The benchmarks are executed on a computer with 8 cores: two Intel Xeon E5310 Quad Core processors and 8 GB RAM running Ubuntu 9.04. The user-specified parameters equal to the clustered results demonstrated in this paper are:

Weighted merge: Yes
Score method: Sum of squared differences
Normalization: Largest average value
Minimum pruning: 6,7%
Minimum overlap: 50%

We measured the total computation time for parallel execution on multiple cores and calculated the speedup gained by comparing to execution on a single core. The speedup plot (Figure S9) shows a close to linear speedup for up to 8 cores, when executing our benchmarks. The benchmark data set consisted of 5920 profiles, 8 tracks and a track length of 52. On the 8-core machine the CATCH algorithm required approximately 57 minutes to finish, illustrating the algorithm capacity for clustering larger data sets within a reasonable running time.

The time spent on alignment of the profiles can be reduced by increasing the percentage minimum overlap of the aligned profiles. Setting the minimum overlap to 100%, requires the shortest of two profiles to be aligned 100%, and for profiles of equal length this corresponds to disallowing alignment. Decreasing the minimum overlap increases the computation time for the similarity score. The added cost of performing alignment of the profiles is less when the execution is running on 8 cores vs. 1 core. Running time as a function of increasing the minimum overlap is shown for 1, 4 and 8 threads in Figure S10.

## References

1. Smith SW (1997) The Scientist & Engineerʼs Guide to Digital Signal Processing. California Technical Pub.

2. Gelder R de, Wehrens R, Hageman JA (2001) A generalized expression for the similarity of spectra: application to powder diffraction pattern classification. Journal of Computational Chemistry 22: 273-289. doi:10.1002/1096-987X(200102)22:3<273::AID-JCC1001>3.0.CO;2-0
